# Procyanidin B2 improves developmental capacity of bovine oocytes via promoting PPARγ/UCP1‐mediated uncoupling lipid catabolism during in vitro maturation

**DOI:** 10.1111/cpr.13687

**Published:** 2024-06-12

**Authors:** Yuwen Luo, Jun Li, Lv Zheng, Yizaitiguli Reyimjan, Yan Ma, Shuaixiang Huang, Hongyu Liu, Guizhen Zhou, Jiachen Bai, Yixiao Zhu, Yidan Sun, Xinhua Zou, Yunpeng Hou, Xiangwei Fu

**Affiliations:** ^1^ State Key Laboratory of Animal Biotech Breeding China Agricultural University Beijing China; ^2^ College of Biological Sciences China Agricultural University Beijing China; ^3^ Department of Reproductive Medicine, Reproductive Medical Center The First Hospital of Hebei Medical University Shijiazhuang Hebei China; ^4^ National Engineering Laboratory for Animal Breeding, Key Laboratory of Animal Genetics, Breeding and Reproduction of the MARA, Beijing Key Laboratory for Animal Genetic Improvement, College of Animal Science and Technology China Agricultural University Beijing China; ^5^ State Key Laboratory of Sheep Genetic Improvement and Healthy Breeding Xinjiang Academy of Agricultural and Reclamation Sciences Shihezi, Xinjiang China

## Abstract

Metabolic balance is essential for oocyte maturation and acquisition of developmental capacity. Suboptimal conditions of in vitro cultures would lead to lipid accumulation and finally result in disrupted oocyte metabolism. However, the effect and mechanism underlying lipid catabolism in oocyte development remain elusive currently. In the present study, we observed enhanced developmental capacity in Procyanidin B2 (PCB2) treated oocytes during in vitro maturation. Meanwhile, reduced oxidative stress and declined apoptosis were found in oocytes after PCB2 treatment. Further studies confirmed that oocytes treated with PCB2 preferred to lipids catabolism, leading to a notable decrease in lipid accumulation. Subsequent analyses revealed that mitochondrial uncoupling was involved in lipid catabolism, and suppression of uncoupling protein 1 (UCP1) would abrogate the elevated lipid consumption mediated by PCB2. Notably, we identified peroxisome proliferator‐activated receptor gamma (PPARγ) as a potential target of PCB2 by docking analysis. Subsequent mechanistic studies revealed that PCB2 improved oocyte development capacity and attenuated oxidative stress by activating PPARγ mediated mitochondrial uncoupling. Our findings identify that PCB2 intricately improves oocyte development capacity through targeted activation of the PPARγ/UCP1 pathway, fostering uncoupling lipid catabolism while concurrently mitigating oxidative stress.

## Graphical abstract

Procyanidin B2 promotes uncoupling lipid catabolism via directly activating peroxisome proliferator‐activated receptor gamma/uncoupling protein 1 pathway, which maintains mitochondrial function and ultimately improves oocyte developmental competence during in vitro maturation.

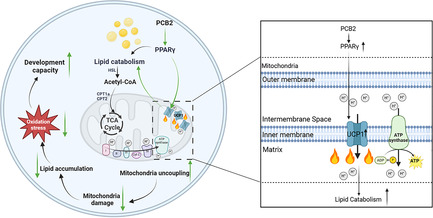

## 1 BACKGROUND

Low quality oocyte is the main reason for poor reproductive outcomes. Oocyte quality and competency are closely linked to metabolic processes, particularly lipid metabolism.^1^, ^2^, ^3^, ^4^ The in vitro maturation (IVM) environment, especially the addition of serum‐like substances, can influence lipid content.^5^, ^6^ Altered lipid levels in this environment would compromise fertility by affecting oocyte quality.^7^, ^8^, ^9^ Research indicates that modulation of lipid metabolism is critical for oocyte maturation and embryo development.^1^, ^10^, ^11^, ^12^, ^13^

Procyanidin B2 (PCB2) is a promising natural compound in regulating cellular lipid metabolism. Previous studies showed that PCB2 ameliorated hepatic steatosis, and exerted beneficial effects through reducing intracellular lipid deposition and ROS production in hepatocytes through autophagy promotion.^14^, ^15^ Additionally, our prior studies demonstrated that PCB2 played a crucial role in maintaining mitochondrial function and oocyte quality under stress conditions.^16^, ^17^ However, a comprehensive investigation on the function of PCB2 in oocytes during IVM remains largely unexplored.

Beyond their role as the cellular powerhouse, mitochondria are also the “hub” implicated in cellular metabolism. Mitochondrial uncoupling, defined as incomplete coupling of mitochondrial oxidative phosphorylation accounts for 25% of the basal metabolic rate.^14^ Previous studies indicated that mitochondrial uncoupling process was intricately intertwined with metabolic regulation.^18^, ^19^ Notably, uncoupling is frequently associated with the modulation of lipid metabolism.^20^, ^21^ For instance, the uncoupling protein 1 (UCP1) has been identified as a mediator of lipid depletion in renal cells during acute kidney injury.^19^

Importantly, peroxisome proliferator‐activated receptors (PPARs) are key transcription factors belonging to the nuclear hormone receptor superfamily and also play a pivotal role in regulating energy metabolism.^22^ Recent studies have demonstrated that PPARγ activation enhances lipid metabolism, thereby reducing the accumulation of lipid.^23^, ^24^, ^25^ Although it is well‐established that UCP1 transcription is regulated by PPARγ,^26^ the specific role of UCP1 and PPARγ in regulating lipid metabolism in oocytes remains a yet unexplored aspect.

Therefore, the present study was conducted to evaluate the impact of PCB2 on bovine oocyte developmental potential, highlighting the specific role of PCB2 in promoting lipid catabolism via PPARγ/UCP1 regulation. Our study will provide novel insights into deciphering the regulatory role of metabolic patterns in oocyte competence acquisition.

## 2 MATERIALS AND METHODS

### 2.1 ETHICS STATEMENT

All procedures were performed according to established ethical guidelines and approved by the Animal Care and Use Committee of China Agricultural University.

### 2.2 Chemicals and antibodies

All chemicals and drugs were purchased from Sigma (St. Louis, MO, USA) unless otherwise indicated. The anti‐rabbit IgG (H + L), F (ab′)_2_ Fragment (Alexa Fluor® 594 Conjugate) antibody, and anti‐SOX2 antibody were purchased from **Cell Signalling Technology** (Danvers, MA, USA). The anti‐CDX2 antibody was purchased from BioGenex (San Francisco, USA). The anti‐Caspase‐3 antibody was purchased from Abcam (Cambridge, UK). The anti‐BAX antibody and anti‐BCL2 antibody were purchased from Beyotime (Beijing, China). The Fluorescein–conjugated Affinipure Goat Anti‐Rabbit IgG (H + L) secondary antibody, anti‐UCP1 antibody, and anti‐PPARγ antibody were purchased from Proteach were purchased from Proteintech (Beijing, China).

### 2.3 Oocyte collection and IVM

Bovine ovaries were collected from a local abattoir and transported to our laboratory within 2–3 h. Cumulus‐oocyte complexes (COCs) were aspirated from 3 to 8 mm follicles using an 18‐gauge needle fitted with a 10 ml disposable syringe. The cellular precipitate was then transferred to a searching dish and COCs with uniform cytoplasm were selected under a stereomicroscope. After that, COCs were transferred to oocyte mature medium (OMM), which was TCM199 containing Earle salts (Gibco, New York, USA), supplemented with Glutamax™ (200 mM, Gibco), sodium pyruvate (100 mM, Gibco), folltropin (0.2 U/ml), estradiol (2 μg/ml), gentamycin (0.01 g/ml), FBS [10% (v/v]) and epidermal growth factor (10 ng/ml), and cultured for 22–24 h at 38.5°C in 5% CO_2_ with humidified air. PCB2 (5 μg/ml), Guanosine diphosphate (GDP, 2 mM, Abcam), and GW9662 (20 μM, MedChemExpress, Shanghai, China) were added in OMM according to the purpose of the experiment.

### 2.4 In vitro fertilization and embryo culture

The in vitro fertilization (IVF) method was described by Hu et al.^27^ After fertilization, oocytes were washed and cultured in in vitro culture (IVC) medium for SOF (Caisson, Utah, USA) supplemented with minimum essential medium–non‐essential amino acids (EAA10 μg/ml), basal medium eagle–EAAs (20 μg/ml), Glutamax™ (200 mM, Gibco), sodium pyruvate (100 mM, Gibco), gentamycin (0.01 g/ml), and BSA (0.2% w/v). Cleavage rates were recorded at 48 h, and blastocyst formation rate was counted on D7 and D8 after IVF (D0), respectively.

### 2.5 Immunofluorescence detection

The immunofluorescence detection method was optimized according to a previous report.^28^ Oocytes or embryos were fixed with 4% paraformaldehyde for more than 1 h and then permeabilized with 0.5% Triton X‐100 at room temperature for 1 h. Followed by blocking in 3% BSA for 1 h at room temperature, oocytes or embryos were incubated with different primary antibodies overnight at 4°C (anti‐BAX, 1:100; anti‐BCL2; 1:100, anti‐CDX2, 1:250; anti‐SOX2, 1:400; anti‐Caspase‐3, 1:200; anti‐UCP1, 1:500; anti‐PPARγ, 1:500). After that, oocytes or embryos were incubated with the corresponding secondary antibody for 1 h at room temperature. Finally, all oocytes or embryos were stained with 4′,6‐diamidino‐2‐phenylindole (DAPI, Vector Laboratories, Burlingame, CA, USA) for 10 min.

Edu (Beyotime), staining was conducted according to the manufacturer's instructions. Briefly, embryos were incubated for 2 h in IVC medium with EdU, then fixed with 4% paraformaldehyde for more than 12 h. Embryos were then permeabilized with 0.5% Triton X‐100 at room temperature for 30 min, and then embryos were incubated with Apollo staining reaction solution for 3 min. Finally, embryos were stained with DAPI for 10 min.

The fluorescent images were taken with laser scanning confocal microscopy (A1 Cell Imaging System; Nikon, Tokyo, Japan) under the same staining procedure and confocal microscopy parameters.

### 2.6 Transmission electron microscopy analysis

After being washed three times with DPBS, oocytes were fixed in 4% paraformaldehyde for 1 h at room temperature, followed by fixation in 2.5% glutaraldehyde for 14 h. Subsequently, the samples were treated with a special osmic acid solution (1% osmic acid and 1.5% potassium ferrocyanide) on ice for 60 min and washed three times with ddH_2_O. Next, the samples were stained with 1% uranyl acetate at room temperature for 1 h. Dehydration was then carried out using a series of ethanol concentrations (50, 70, 80, 90, and 100%). The samples underwent two cycles of soaking in propylene oxide and were subsequently permeated with 812 resin and propylene oxide overnight at room temperature. Following embedding, the samples were serially sectioned with an automated microtome to a thickness of 70 μm, and sections with the largest oocyte diameter were affixed onto copper meshes. The sections were stained with uranium dioxyacetate and examined under transmission electron microscopy (TEM) at selected magnifications of 7000× and 20,000×. The number of lipid droplet/mitochondria, lipid droplet/mitochondrial area, abnormal mitochondrial percentage, and mitochondrial electron density were evaluated for each image in the fixed area of oocytes.

### 2.7 Targeted metabolomics analysis

A total amount of 0.05 ml OMM medium were collected and mixed with 150 μl MeOH, 200 μl MTBE, and 50 μl 36% phosphoric acid/water (precooled at −20°C). The sample was homogenized by vortex (2500 rpm/min) for 3 min and centrifuged at 12,000 rpm/min for 5 min at 4°C. Then, 200 μl supernatant was aspirated into a new centrifuge tube. After drying, 300 μl methanol solution of 15% boron trifluoride was added into the tube. Samples were homogenized by vortex (2500 rpm/min) for 3 min and then kept in the oven at 60°C for 30 min. When the sample was cooling down to room temperature, 500 μl *n*‐hexane and 200 μl saturated sodium chloride solution were accurately added. After vortexing for 3 min and centrifugation at 12,000 rpm for 5 min at 4°C, 100 μl *n*‐hexane layer solution was transferred for further GC–MS analysis.

The saccharides in the OMM were also measured. Briefly, 50 μl of sample was added with 500 μl methanol: isopropanol: water (3:3:2 V/V/V). The mixtures were vortexed for 3 min, ultrasonicated for 30 min, and subsequently centrifuged at 14,000 rpm at 4°C for 3 min. Then, 50 μl of the supernatant was mixed with 20 μl internal standard (Ribitol, 1000 μg/ml) and evaporated under nitrogen gas stream. The evaporated sample was transferred to the lyophilizer for freeze‐drying. The residue was used for further derivatization. After that, the mixture was incubated at 37°C for 2 h. Then, 100 μl of BSTFA was added to the mixture and kept at 37°C for 30 min after vortex‐mixing. Agilent 8890 gas chromatograph coupled to a 5977B mass spectrometer with a DB‐5MS column (30 m length × 0.25 mm i.d. × 0.25 μm film thickness, J&W Scientific, USA) was employed for GC–MS analysis of saccharides.

### 2.8 Lipid staining

Nile Red (MedChemExpress) staining was used to measure the lipid content in oocytes. Samples from each treatment group were fixed in 4% paraformaldehyde for more than 30 min at room temperature. Then, oocytes were incubated with Nile Red work solution (3 mM) at room temperature for 25 min. Finally, oocytes were observed with a laser confocal microscope (A1 Cell Imaging System; Nikon, Tokyo, Japan). Fluorescence intensity was analysed using NISElements AR software (Nikon Instruments, Tokyo, Japan).

### 2.9 Live cell imaging

Live cell imaging was conducted as previously reported by Meng et al.^29^ with minor modifications. In short, MII oocytes were incubated for 45 min with 2‐NBDG (200 μM, Apexbio, Houston, TX, USA), 2′,7′‐dichlorofluorescin diacetate (2′,7′‐DCFHDA) (1 mM, Beyotime), ThiolTracker Violet (10 μM, Invitrogen, Carlsbad, CA, USA), or Mito‐SOX (5 μM, Invitrogen) at 38.5°C to determine glucose uptake, ROS, GSH in oocyte and ROS in the mitochondria, respectively. Then, the oocytes were washed with a washing buffer to remove surface fluorescence and observed using a fluorescence microscope (Olympus IX73). The fluorescence of each oocyte was analysed by EZ‐C1Free‐Viewer (Nikon, Tokyo, Japan).

Oocytes were incubated with 100 nM TMRM (Invitrogen) in 100 μM working solution at 38.5°C in 5% CO_2_ for 30 min. MMP was calculated by the TMRM fluorescence intensity. The oocytes were then washed three times with DPBS and examined using a laser confocal microscope (A1 Cell Imaging System; Nikon, Tokyo, Japan) with an excitation wavelength of 594 nm. NISElements AR software was used to assess the fluorescence intensity (Nikon Instruments, Tokyo, Japan).

Mitochondrial‐targeted florescent dye Mito Thermo Yellow (MTY) was used to detect mitochondrial temperature. MTY fluorescence intensity was negatively correlated with mitochondrial temperature.^30^ Oocytes were incubated with 0.5 μM MTY for 15 min. After that, oocytes were observed using a fluorescence microscope (Olympus IX73). The software of the EZ‐C1 Free‐Viewer (Nikon) Microscope was used to analyse fluorescence.

### 2.10 Assay of ATP Content

The level of ATP in each oocyte was measured using an Enhanced ATP Assay Kit (Beyotime) according to the method of the previous study.^31^ ATP standards with different concentration gradients were prepared (0, 2, 4, 8, 16, 32 pmol). For each group, 8–10 denuded oocytes were collected in a 0.2 ml centrifuge tube containing 20 μl lysis buffer on ice. Oocytes were homogenized by vortexing until lysis. ATP assay buffer was added to 96‐well plates and equilibrated for 3–5 min at room temperature. Then, standard solutions and ATP detection diluent were injected into each well, and luminescence activity was measured immediately using a luminometer (Infinite F200, Tecan). ATP content was calculated using a standard curve. The total amount of ATP was divided by the number of oocytes in each sample to obtain the mean content per oocyte (pmol/oocyte).

### 2.11 Quantitative real‐time PCR

After maturation, a total of 30 MII oocytes per replicate was collected from different groups. RNA was extracted from the sample using QIAGEN RNeasy Mini Kit (Qiagen, Valencia, CA, and the USA), and then it was reversed to cDNA using a QuantiTect Reverse Transcription Kit (Qiagen). All Primers used in this assay are designed using the NCBI Primer as shown in Table 1. Primers were tested for efficiency to ensure their specificity. Quantitative real‐time (qRT‐PCR) was performed using an ABI 7500 real‐time PCR instrument and a Fast 96‐well Thermal Cycler (Applied‐Biosystems, Foster City, CA, USA). Relative mRNA levels of target genes were calculated using the 2^−ΔΔCt^ method with *ACTB* and *H2AFZ* as the reference genes according to previous reports.^32^

**TABLE 1:** Primer sequences used for quantitative real‐time PCR.

Gene	Primer sequence (5′–3′)	NCBI reference sequences
ATP5F1E	F: TGGACTCAGCTACATCCGATAC R: AGTCTTCATGGCGTTTGCTT	NM_001143741.1
CAT	F: CAGGGTGGGGCTCCAAATTA R: GAAAGTCCGCACCTGAGTGA	NM_001035386
CASPASE‐3	F: TACTTGGGAAGGTGTGAGAAAACTAA R: AACCCGTCTCCCTTTATATTGCT	NM_001077840
CPT1A	F: TCCTGGTGGGCTACCAATTA R: TGCGTCTGTAAAGCAGGATG	NM_001304989
CPT1B	F: TCTCCAGCAAGTTCTCCAGC R: ATCTCTCCAGCCCTTAGCCA	NM_001034349.2
CPT2	F: TTTGGCATTGGGTACTCCGT R: TATGCTGGTGAAACAGAGGCT	NM_001045889.2
G6PDH	F: GGTACTGGTGGCAAGTCCAT R: GCCATCCAACCACTCAGTCT	NM_001244135.2
GLUT1	F: CCAAGGATCTCTCAGAGCACAG R: TTCTTCTGGACATCACTGCTGG	NM_174602.2
GPX1	F: CTGAAGTACGTCCGACCAGG R: GTCGGTCATGAGAGCAGTGG	NM_174076.3
HSL	F: CCTCGTGGCTCAACTCCTTCTTG R: TCTGTTGTGTCACTGCTGTTCCTG	NM_001080220.1
SDHB	F: AAACTGGACGGGCTCTAT R: AGGTTGCCATCATCTTCTT	NM_001040483.1
SOD2	F: GGATCCCCTGCAAGGAACAA R: TGGCCTTCAGATAATCGGGC	NM_201527
UCP1	F: AGGTGTCCTGGGAACAATCA R: ACACTGCCTGCTTACCTTCTT	NM_001166528.1
UCP2	F: CGACGTGGTCAAGACGAGAT R: AGGAGGGCATGAACCCTTTG	NM_001033611.2
ACTB	F: ATTTTGAATGGACAGCCATC R: TGTACAGGAAAGCCCTGACT	NM_173979.3
H2AFZ	F: CTCACCGCAGAGGTACTTGAATT R: AGTCCAATTCTTCATCTCCACGA	NM_174809.2

Abbreviations: *ACTB*, actin beta; *ATP5F1E*, *ATP synthase F1 subunit epsilon*; *CASPASE‐3*, caspase 3; *CAT*, catalase; *CPT1A*, carnitine palmitoyltransferase 1A; *CPT1B*, carnitine palmitoyltransferase 1B; *CPT2*, carnitine palmitoyltransferase 2; *G6PDH*, glucose‐6‐phosphate dehydrogenase; *GLUT1*, glucose transporter 1; *GPX1*, glutathione peroxidase 1; *H2AFZ*, H2A.Z variant histone 1; *HSL*, lipase E, hormone sensitive type; *SDHB*, succinate dehydrogenase complex iron sulphur subunit B; *SOD2*, superoxide dismutase 2; *UCP1*, uncoupling protein 1; *UCP2*, uncoupling protein 2.

### 2.12 Molecular docking

The crystal structure of PPARγ (PDB ID: 6K0T) was downloaded from the Protein Data Bank (https://www.rcsb.org/). Molecular Docking studies were performed using Autodock vina.^33^ The docking results were analysed in clusters, and the model with the lowest binding energy was selected as the proposed position for PCB2 and then visualized with Pymol (The PyMOL Molecular Graphics System).

### 2.13 Statistical analysis

All experiments were performed with at least three biological replicates, and all values are presented as mean ± standard error (SEM). Data were analysed and plotted using GraphPad Prism 8. Student's *t* test was used for comparison between two independent samples (*P < 0.05, **P < 0.01, and ***P < 0.001 for significant differences). The comparison of means among the multiple groups of data was analysed by one‐way ANOVA. Tukey's multiple comparisons and different superscripts indicate significant differences (*P* < 0.05). The metabolites with a fold change ≥1.2 or ≤0.83 and variable importance in the projection value ≥1 were considered as differentially abundant metabolites. Univariate analysis was also another way of obtaining information from the metabolome data,^34^ and comparisons using a threshold of fold change ≥1.2 or ≤0.83.

## 3 RESULTS

### 3.1 PCB2 during IVM supplementation improves oocyte development potential

**FIGURE 1:**
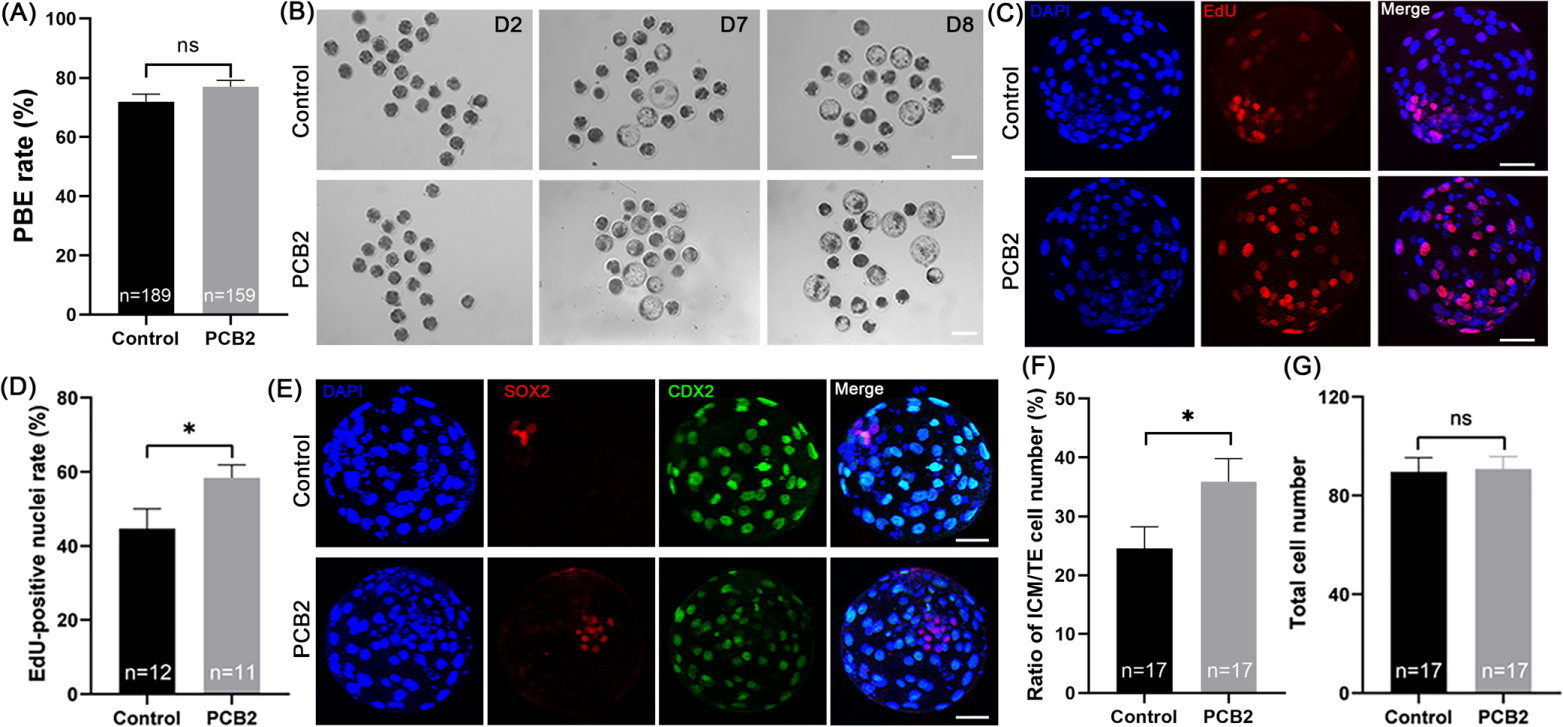
PCB2 supplementation improves oocyte developmental capacity. (A) The rate of PBE in control and PCB2‐treated oocytes. (B) Representative images of early embryos derived from in vitro fertilized oocytes. Scale bar, 100 μm. (C) Representative images of blastocyst stained with EdU and DNA. Scale bar, 50 μm. (D) Statistical analysis of the percentage of EdU‐positive cells. (E) Representative images of blastocysts stained with DNA, CDX2, and SOX2. Scale bar, 50 μm. (F) Statistical analysis of the ratio of ICM/TE. (G) Statistical analysis of total cell number. “n” represents the number of cells utilized in this study. All experiments were conducted in triplicate, and data are presented as mean ± SEM. ns = non significance, *, *P* < 0.05, **, *P* < 0.01.

We conducted IVF to investigate the impact of PCB2 on oocyte maturation and subsequent embryo development. The addition of PCB2 significantly increased blastocyst formation on D7 and D8 during oocyte maturation, whereas it did not affect polar body extrusion or cleavage rates (Figure 1A,B, Table 2). Moreover, PCB2 supplementation notably enhanced blastocyst proliferation capacity (Figure 1C,D). As shown in Figure 1E–G, the blastocyst quality was significantly improved after PCB2 treatment indicated by CDX2 (a cell lineage‐specific marker for trophectoderm [TE]) and SOX2 (a cell lineage‐specific marker for inner cell mass [ICM]) staining. The ratio of ICM to TE was significantly increased in the PCB2 group. Thus, our findings indicated that PCB2 supplementation significantly improved oocyte subsequent developmental capacity.

**TABLE 2:** Subsequent development analysis of oocytes following IVF.

	Control	PCB2
Total COCs (*n*)	312	278
Cleaved embryos (%)	237 (76.51 ± 2.99%)	211 (76.62 ± 3.08%)
D7 blastocysts (% from cleaved)	42 (18.44 ± 2.61%)	58 (28.56 ± 2.82%)**
D8 blastocysts (% from cleaved)	56 (24.55 ± 2.17%)	70 (34.57 ± 3.90%)*

*Note*: “*n*” represents the cell number used in this experiment. All experiments were performed in triplicate and the data represent the mean ± SEM.

* *P* < 0.05.

** *P* < 0.01.

### 3.2 PCB2 ameliorates oxidative stress in oocytes

**FIGURE 2:**
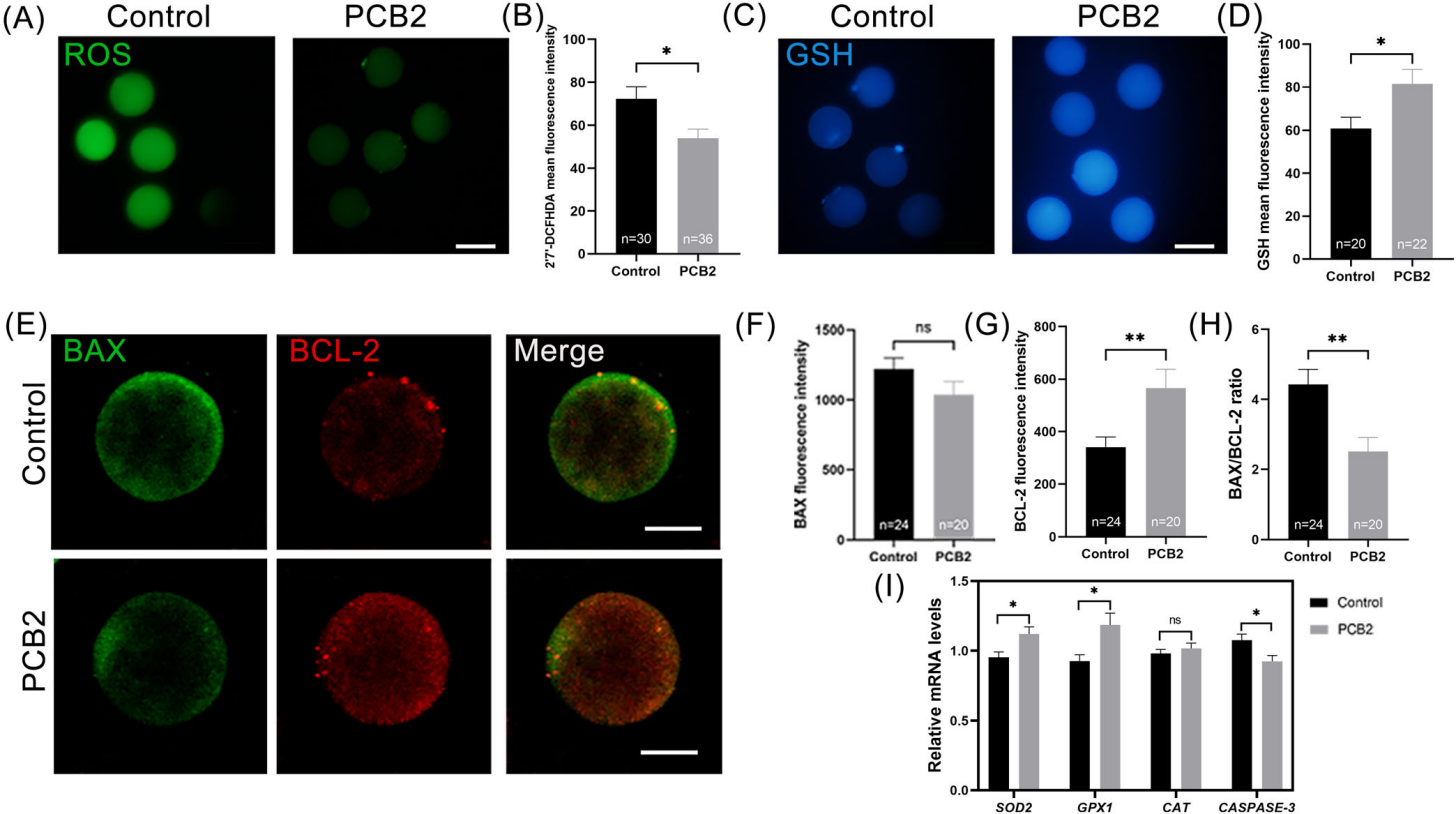
Effect of adding PCB2 on oxidative stress status and apoptosis in oocytes. (A) Representative images of MII oocytes stained with ROS. Scale bar, 100 μm. (B) Statistical analysis of ROS fluorescence intensity. (C) Representative images of MII oocytes stained with GSH. Scale bar, 100 μm. (D) Statistical analysis of GSH fluorescence intensity. (E) Representative images of MII oocytes stained with BAX and BCL‐2. Scale bar, 50 μm. (F) Statistical analysis of BAX fluorescence intensity. (G) Statistical analysis of BCL‐2 fluorescence level. (H) Statistical analysis of the ratio of BAX/BCL‐2. (I) Statistical analysis of relative expression levels of *SOD2*, *GPX1*, *CAT*, and *CASPASE‐3* in MII bovine oocyte. “*n*” represents the cell number used in this experiment. All experiments were performed in triplicate and data were represented as mean ± SEM. ns = non significance, *, *P* < 0.05, **, *P* < 0.01.

Oxidative stress caused by excessive ROS is the primary reason for the decreased quality of IVC oocytes.^8^ Then, we evaluated whether PCB2 would attenuate oxidative stress in IVM oocytes. Our results revealed that PCB2 supplementation during maturation notably lowered intracellular ROS levels and increased GSH levels significantly (Figure 2A–D). Additionally, upregulated expression levels of *SOD2* and *GPX1* were found in oocytes treated with PCB2 (Figure 2I). BAX/BCL2 ratio was thought to be a key factor of apoptosis initiation.^35^ Our results indicated that PCB2 treatment significantly elevated BCL2 levels and consequently reduced the BAX/BCL2 ratio in oocytes (Figure 2E–H). Notably, PCB2 supplementation mitigated oocyte apoptosis during maturation, as evidenced by reduced *CASPASE‐3* expression (Figure 2I).

### 3.3 PCB2 exhibits catabolic preference for lipids during oocyte maturation

**FIGURE 3:**
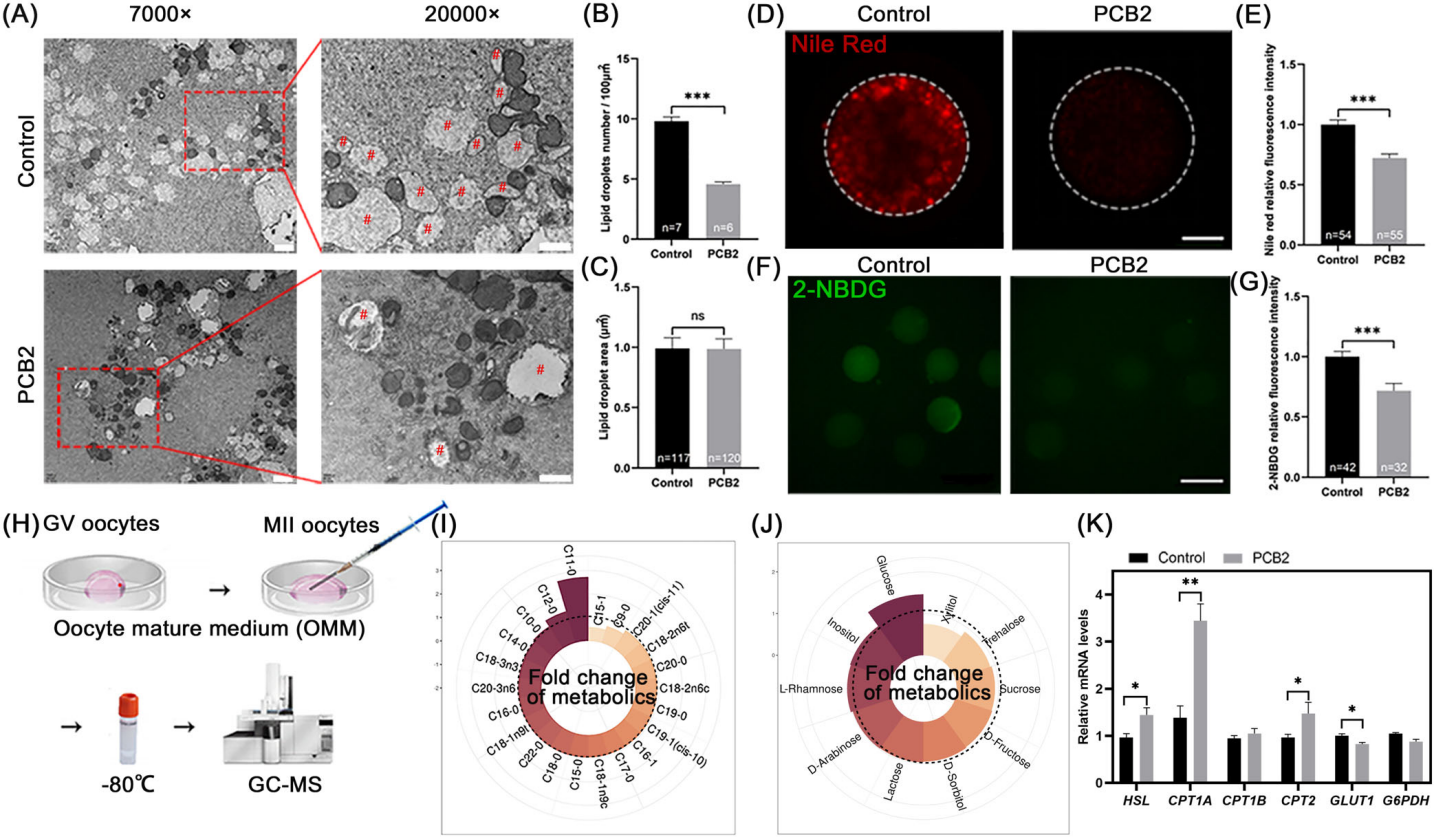
Effect of adding PCB2 on lipid metabolism and glucose uptake. (A) Representative images of lipid droplets in MII oocytes by TEM. 7000×, scale bar, 2 μm, 20,000×, scale bar, 1 μm. Red pound key (#) represents the lipid droplet. (B) Quantification analysis of the lipid droplet number per 100 μm^2^. (C) Quantification analysis of the mitochondrial area in oocytes. (D) Representative images of MII oocytes stained with Nile Red. Scale bar, 20 μm. (E) Statistical analysis of Nile Red fluorescence intensity. (F) Representative images of MII oocytes stained with 2‐NBDG. Scale bar, 100 μm. (G) Statistical analysis of 2‐NBDG fluorescence intensity. (H) Schematic diagram of metabolomic sample collection. (I) Nightingale rose chart represents the fold change of lipids in the medium between Control and PCB2 groups. Black dotted box, fold change = 1. (J) Nightingale Rose Chart represents the fold change of carbohydrates in the medium between Control and PCB2 groups. Black dotted box, fold change = 1. (K) Statistical histogram analysis of relative expression levels of *CPT1a*, *CPT1b*, *CPT2*, *HSL GLUT1*, and *G6PDH*, in MII oocytes. “*n*” represents the cell droplet number used in this experiment. All experiments were performed in triplicate and the data were represented as mean ± SEM. *, *P* < 0.05, **, *P* < 0.01, ***, *P* < 0.001.

Lipid accumulation can lead to oxidative stress and apoptosis during IVM.^4^ Based on this, we hypothesized that PCB2 could mediate lipid metabolism to alleviate the imbalance of ROS and oxidative stress in oocytes. To test the assumption, we evaluated oocyte lipid content. Initially, using TEM, we observed that PCB2 supplementation decreased the amounts of lipid droplets in oocytes, as counted by the number in 100 μm^2^ (Figure 3A–C). Furthermore, Nile Red staining indicated a significant reduction in intracellular lipid levels with PCB2 supplementation (Figure 3D,E).

Moreover, we analysed the metabolic profiles of OMM from each group as shown in Figure 3H, and a total of 22 fatty acids were detected. The top four components were consistent between PCB2 treatment and the control groups, namely palmitic acid (C16‐0), stearic acid (C18‐0), cis‐9‐octadecenoic acid (C18‐1), and linoleic acid (C18‐2). Notably cis‐10‐pentadecenoic acid (C15‐1) levels were markedly reduced in the PCB2 group (Table S1, Figure 3I). Gene expression analysis confirmed that PCB2 increased fatty acid β‐oxidation, as *CPT1a*, *CPT2*, and *HSL* levels were significantly elevated (Figure 3K), supporting enhanced lipid catabolism.

Given that carbohydrate is another kind of crucial energy source for oocyte development,^36^ we investigated carbohydrate composition in OMM (Figure 3H). Among the identified 10 kinds of carbohydrates, glucose was the compound with the highest content. Multivariate analysis revealed that xylitol level was significantly decreased and glucose content exhibited the most pronounced alteration in oocytes treated with PCB2 (Table S2, Figure 3J). To validate these findings, we used 2‐NBDG to examine the uptake of glucose in oocytes. As shown in Figure 3F,G, PCB2 supplementation markedly reduced glucose uptake by oocytes compared to the controls. Moreover, declined mRNA expression of the glucose transporter *GLUT1* was discovered in PCB2‐treated oocytes (Figure 3K). However, PCB2 had no significant impact on the mRNA expression of *G6PDH* (Figure 3K). These results implied that PCB2 treatment shifted oocyte metabolism towards lipid utilization during IVM.

### 3.4 PCB2 improves mitochondrial function by promoting uncoupling process

**FIGURE 4:**
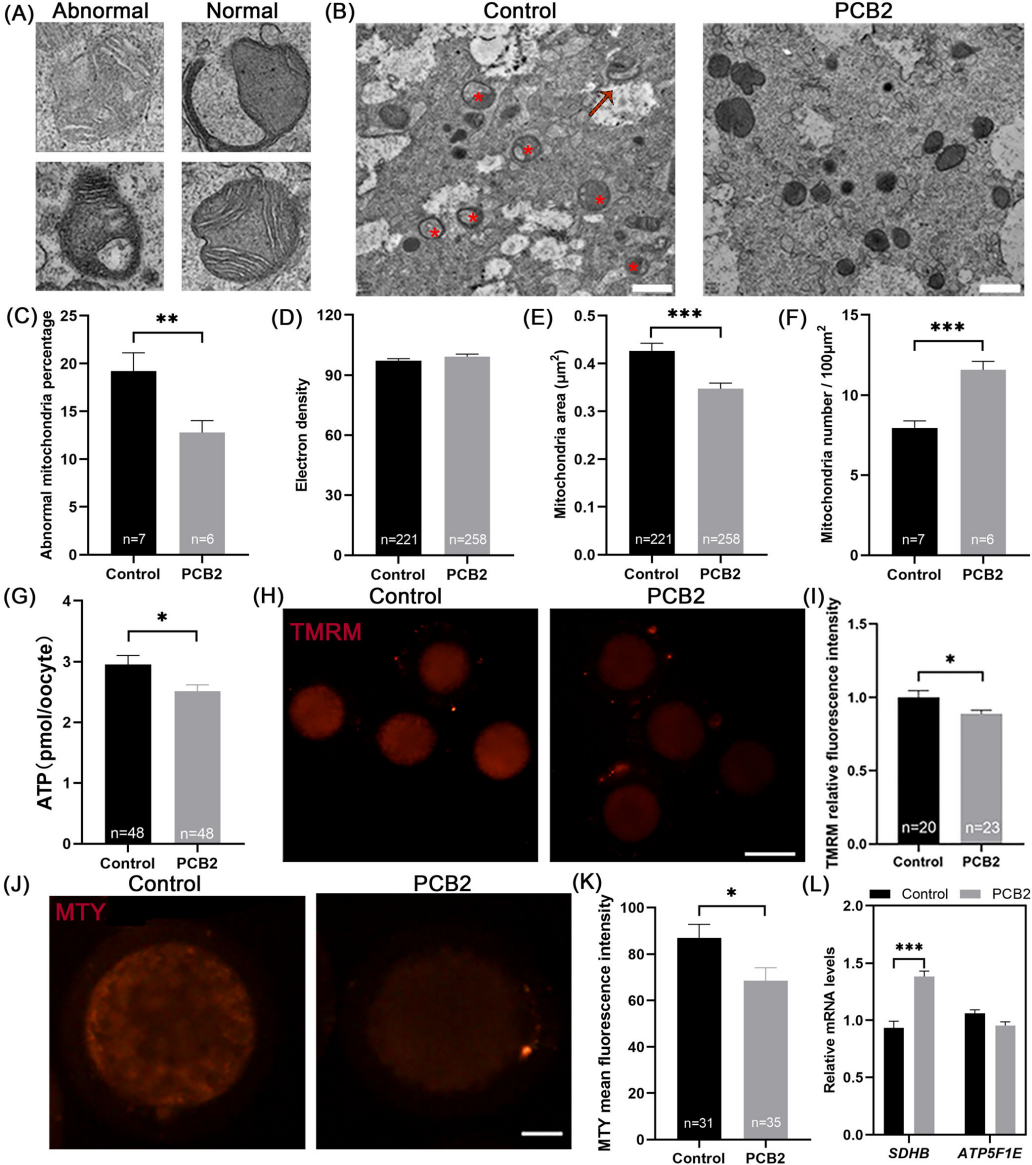
Effect of adding PCB2 on mitochondrial function in oocytes. (A) Mitochondria ultrastructure observed in TEM images of oocytes. The abnormal mitochondria are characterized by a non‐uniform matrix (up‐left) and loss of cristae (down‐left). The normal mitochondrial structure included cap‐shaped (up‐right) and typical spherical with cristae (down‐right). (B) Representative images of mitochondria morphology and structure in Control and PCB2 oocytes by TEM. Red arrow and red star (*) represented cap‐shaped mitochondria and abnormal mitochondria respectively. Scale bar, 2 μm. (C) Quantification analysis of abnormal mitochondria percentage per 100 μm^2^ in Control and PCB2‐treated oocytes. (D) Quantification analysis of the mitochondrial electron density in Control and PCB2 groups of oocytes. (E) Quantification analysis of the mitochondrial area in Control and PCB2 groups of oocytes. (F) Quantification analysis of the mitochondrial number per 100 μm^2^ in Control and PCB2‐treated oocytes. (G) Statistical analysis of ATP content level. (H) Representative images of MII oocytes stained with TMRM. Scale bar, 100 μm. (I) Statistical analysis of MMP fluorescence intensity. (J) Representative images of MII oocytes stained with MTY. Scale bar, 20 μm. (K) Statistical analysis of MTY fluorescence intensity. (L) Statistical analysis of relative expression levels of *SDHB and ATP5F1E* in MII oocytes. “*n*” represents the cell number used in this experiment or mitochondria. All experiments were performed in triplicate and the data were represented as mean ± SEM. ns = non significance, *, *P* < 0.05, **, *P* < 0.01, ***, *P* < 0.001.

Mitochondria are the main sites of lipid metabolism, and the regulation of lipid metabolism is highly related to mitochondrial function.^19^, ^35^ To understand the effect of PCB2 on mitochondrial function, we first analysed mitochondrial ultrastructure in the oocytes by TEM. As expected, mitochondrial abnormalities, such as swelling, vacuolization, loss of mitochondrial cristae and lack of electron density were observed in IVM oocytes, and PCB2 would significantly reduce structural abnormalities in mitochondria (Figure 4A–E). Additionally, the result showed that mitochondrial number was significantly increased in oocytes after PCB2 treatment (Figure 4F).

We then tried to clarify the underlying relationship between lipid metabolism and mitochondria function. Through β‐oxidation, lipids could generate acetyl‐CoA into the tricarboxylic acid cycle (TCA).^37^ Accompanied by TCA, the electron transfer chain could transport protons across the inner mitochondrial membrane and create an electrochemical gradient known as MMP, which was necessary for ATP synthase to produce ATP.^38^ Interestingly, the results showed that ATP content and MMP level were both dramatically reduced after PCB2 treatment (Figure 4G–I). Moreover, *SDHB* expression was significantly increased, whereas the expression of *ATP5F1E* was not altered (Figure 4L). To explore the mechanism underlying the seemingly contradictory phenomenon between improved mitochondrial function and declined ATP content in PCB2‐treated oocytes, the thermogenic state condition of mitochondria was further determined. As a temperature‐sensitive fluorescence probe, MTY was negatively associated with temperature changes. As shown in Figure 4J,K, the temperature of mitochondria was increased in the PCB2‐treated group. Therefore, our results indicated that PCB2 could promote mitochondrial uncoupling process in oocytes, and dissipate energy as heat.

### 3.5 PCB2 regulates lipid catabolism via UCP1‐mediated mitochondria uncoupling

**FIGURE 5:**
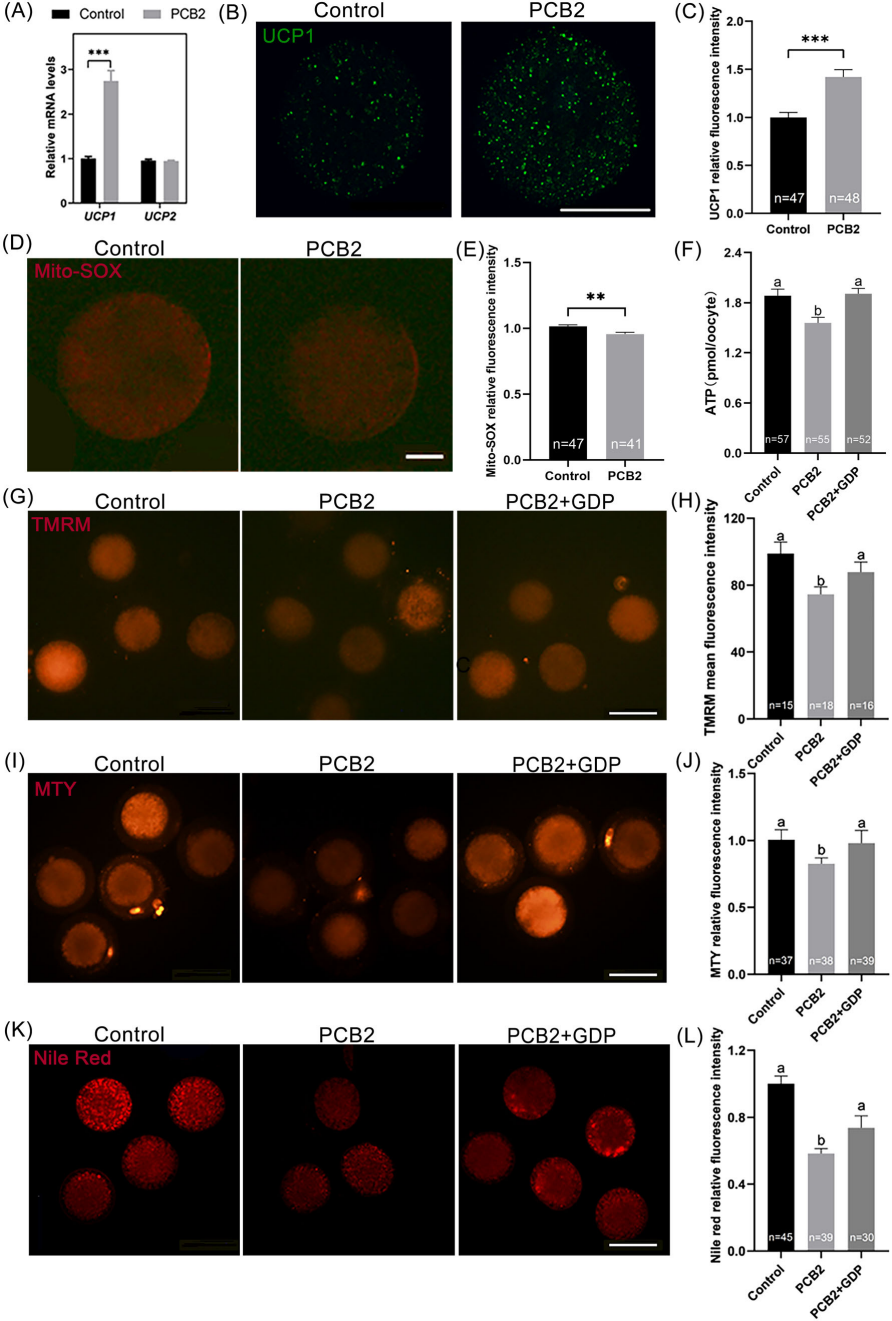
UCP1 antagonist abrogated the positive effects of PCB2 on mitochondrial uncoupling lipid catabolism. (A) Statistical analysis of relative expression levels of *UCP1*, and *UCP2* in MII oocytes. (B) Representative images of MII oocytes stained with UCP1. Scale bar, 50 μm. (C) Statistical analysis of UCP1 fluorescence intensity. (D) Representative images of MII oocytes stained with Mito‐SOX. Scale bar, 20 μm. (E) Statistical analysis of Mito‐SOX fluorescence intensity. (F) Statistical analysis of ATP content. (G) Representative images of TMRM fluorescence intensity. Scale bar, 100 μm. (H) Statistical analysis of TMRM fluorescence intensity. (I) Representative images of MTY fluorescence intensity. Scale bar, 100 μm. (J) Statistical analysis of MTY fluorescence intensity. (K) Representative images of Nile Red fluorescence intensity. Scale bar, 100 μm. (L) Statistical analysis of Nile Red fluorescence intensity. “*n*” represents the cell number used in this experiment. All experiments were performed in triplicate and the data were represented as mean ± SEM. ns = non significance, *, *P* < 0.05, **, *P* < 0.01, ***, *P* < 0.001. Different superscripts within columns indicate significant differences (*P* < 0.05).

Mitochondria regulate energy production and heat generation via uncoupling proteins (UCPs).^39^, ^40^ To understand the impact of PCB2 on mitochondrial thermogenesis, we examined UCPs expression. Notably, *UCP1* mRNA level was significantly increased after PCB2 treatment (Figure 5A). Consistently, the fluorescent signal of UCP1 was also elevated significantly (Figure 5B,C). It was reported that UCPs played a role in mitochondrial superoxide production.^41^, ^42^ In our study, PCB2 treatment led to a significant reduction in mitochondrial ROS levels, as indicated by Mito‐SOX™ staining (Figure 5D,E). This further suggested that PCB2 would regulate mitochondrial thermogenesis and mitigate oxidative stress through UCP1 modulation.

GDP is a well‐recognized potent inhibitor of UCP1.^43^ To further investigate the impact of UCP1 on lipid metabolism, we experimentally inhibited UCP1 with GDP. Interestingly, we found that the PCB2‐induced mitochondrial uncoupling process was suppressed after GDP supplementation. As shown in Figure 5F–L, with the combination treatment of PCB2 and GDP, oocytes showed increased MMP and ATP levels, concomitant with noteworthy reductions in mitochondrial temperature and lipid metabolism. These findings suggested that UCP1‐mediated mitochondrial uncoupling played a crucial role in enhancing lipid catabolism and alleviating oxidative stress in oocytes.

### 3.6 PCB2 exerts active effects by directly interaction with PPARγ

**FIGURE 6:**
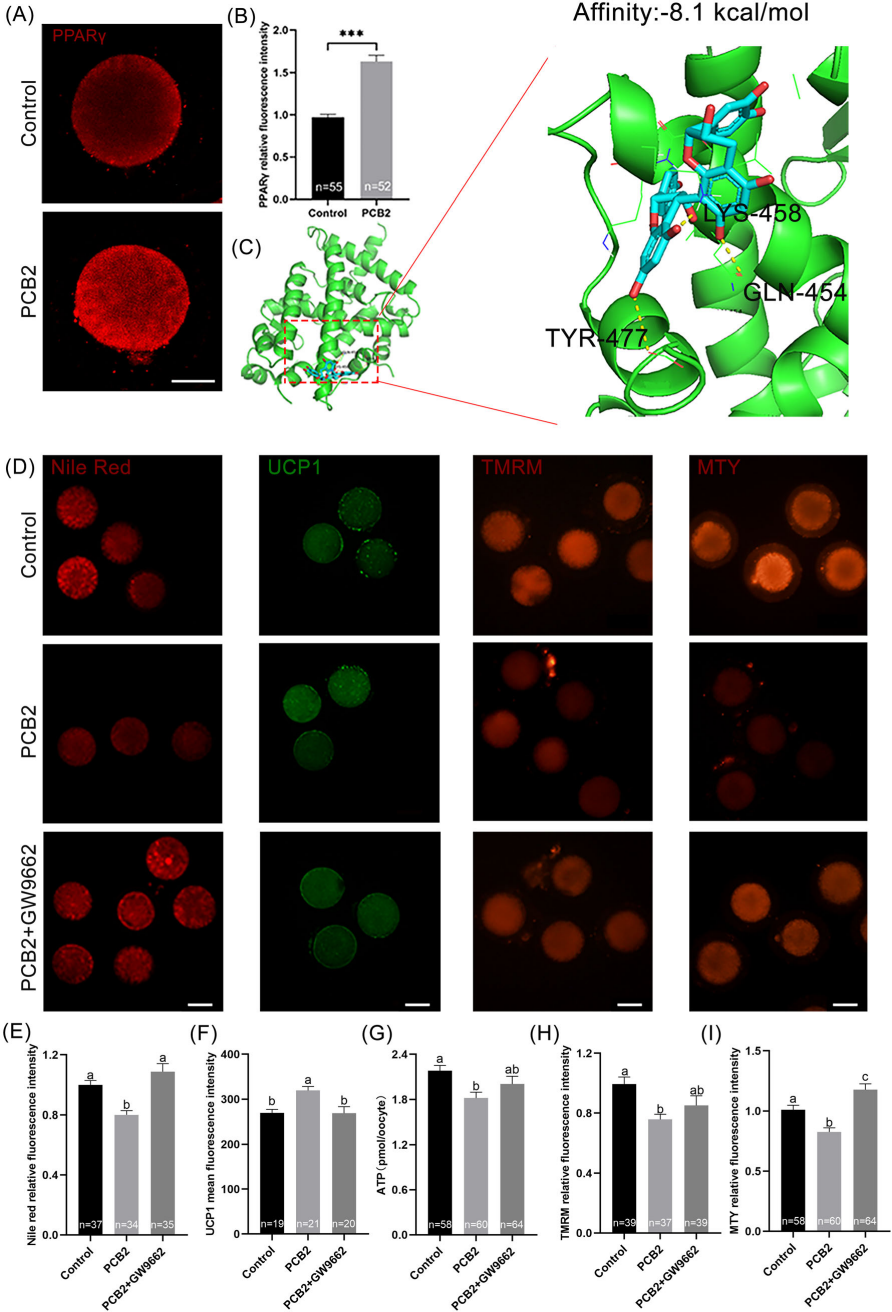
PCB2 promotes mitochondrial uncoupling via PPARγ activation. (A) Representative images of MII oocytes stained with PPARγ. Scale bar, 20 μm. (B) Statistical analysis of PPARγ fluorescence intensity. (C) Representative images of predicted binding conformation of PCB2 with PPARγ model. Yellow dotted line represents hydrogen bonds. (D) Representative images of MII oocytes stained with TMRM, MTY, Nile red, and UCP1. Scale bar, 50 μm. (E) Statistical analysis of Nile red fluorescence intensity. (F) Statistical analysis of UCP1 fluorescence intensity. (G) Statistical analysis of ATP content level. (H) Statistical analysis of TMRM fluorescence intensity. (I) Statistical analysis of MTY fluorescence intensity. “*n*” represents the cell number used in this experiment. All experiments were performed in triplicate and the data were represented as mean ± SEM. ns = non significance, *, *P* < 0.05, **, *P* < 0.01, ***, *P* < 0.001. Different superscripts within columns indicate significant differences (*P* < 0.05).

PPARγ is a key mediator involved in lipid metabolism and thermogenesis.^41^ Based on this, we hypothesized that PCB2 might potentially activate PPARγ to regulate UCP1‐mediated mitochondrial uncoupling. Our results showed that the mRNA and the fluorescent signal of PPARγ were increased in oocytes treated with PCB2 (Figure 6A,B, S1). Molecular docking was also employed to assess possible binding mechanisms of PPARγ with PCB2. As shown in Figure 6C, PCB2 could fit into the possible ligand binding site reported in the literature,^42^ since the hydrogen bonds were formed at GLN454, LYS 458, and TYR477 with strong binding activity (binding energy: −8.1 kcal/mol). Next, a potent PPARγ inhibitor, GW9662, was applied to determine whether PPARγ was directly involved in PCB2‐mediated oocyte mitochondrial uncoupling. As expected, following GW9662 treatment, the function of PCB2 in UCP1 activation and lipid catabolism was completely abolished (Figure 6D–F). Moreover, we found that GW9662 would nullify the beneficial effects of PCB2 on mitochondrial function, for that changes in MMP, ATP content, and mitochondrial temperature induced by PCB2 were all reversed after GW9662 treatment (Figure 6D,G–I). These results further support the notion that PPARγ is a potential drug target of PCB2 in oocytes and suggest that PCB2 regulates lipid deposition by modulating the mitochondrial uncoupling through the PPARγ/UCP1 pathway.

**FIGURE 7:**
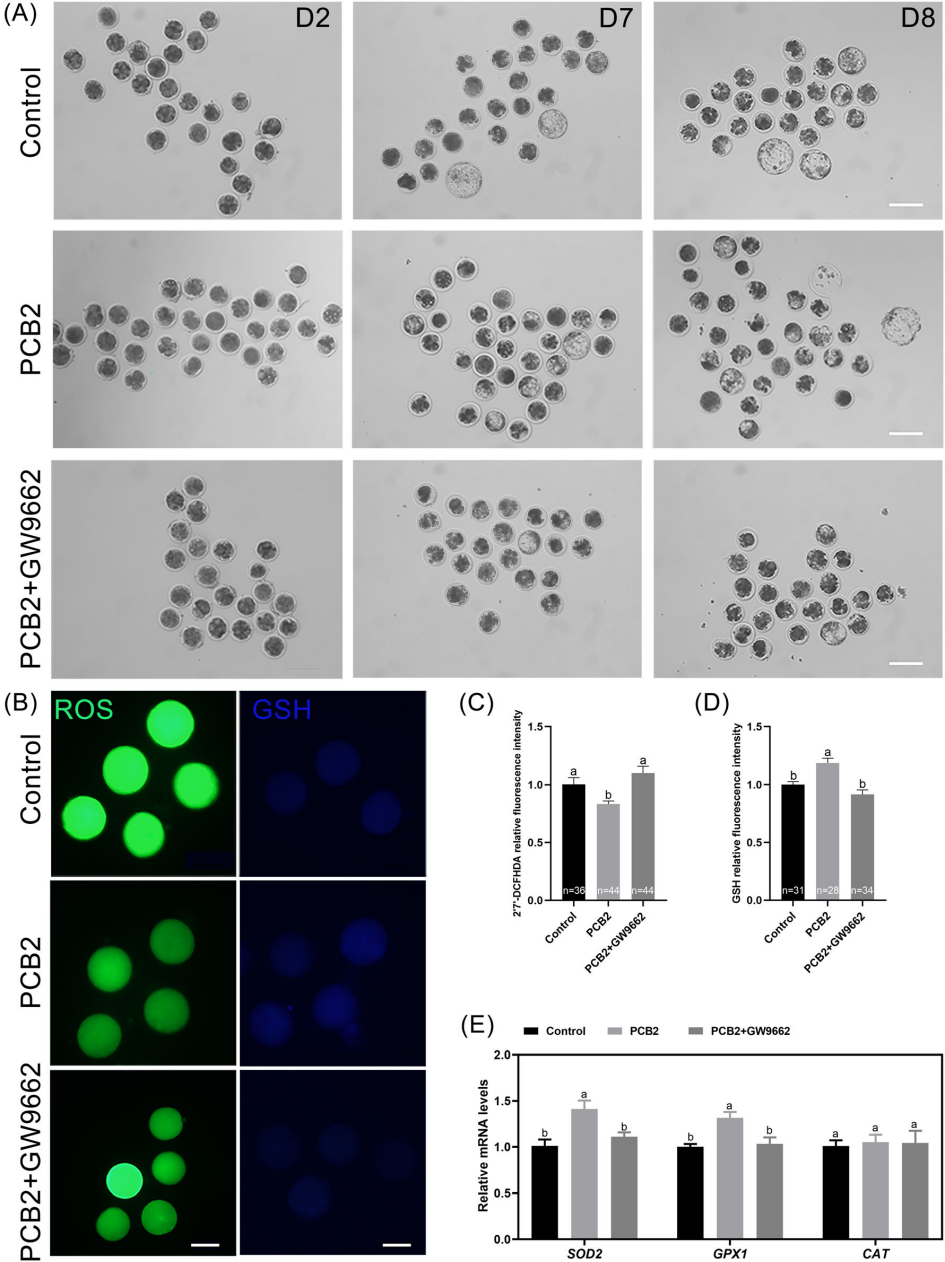
PCB2 exerts protective functions via PPARγ activation. (A) Representative images of early embryos derived from in vitro fertilized oocytes. Scale bar, 100 μm. (B) Representative images of MII oocytes stained with ROS and GSH. Scale bar, 50 μm. (C) Statistical analysis of ROS fluorescence intensity. (D) Statistical analysis of GSH fluorescence intensity. (E) Statistical analysis of relative expression levels of *SOD2*, *GPX1*, and *CAT* in MII oocyte. All experiments were performed in triplicate and the data were represented as mean ± SEM. Different superscripts within columns indicate significant differences (*P* < 0.05).

We also found that the blastocyst rate was significantly reduced in oocytes treated with GW9662 when compared to that of the PCB2 treatment (Figure 7A, Table 3). Additionally, we investigated the role of PCB2 in PPARγ/UCP1 pathways‐mediated redox homeostasis. We found that GW9662 treatment attenuated the impact of PCB2 on oxidative stress, as evidenced by elevated ROS levels and diminished GSH content (Figure 7B–D). Correspondingly, GW9662 nullified alterations in the expression levels of oxidation‐related genes (Figure 7E). These results collectively underscore the pivotal role of PCB2 in enhancing the antioxidant defence system through the PPARγ/UCP1 pathway to improve oocyte development capacity.

**TABLE 3:** Effect of GW9662 on subsequent development of oocytes following IVF.

	Control	PCB2	PCB2 + GW9662
Total COCs (*n*)	118	111	121
Cleaved embryos (%)	87 (73.71 ± 1.73%)^ab^	82 (74.19 ± 2.15%)^a^	81 (67.48 ± 4.17%)^ab^
D7 blastocysts (% from cleaved)	17 (19.72 ± 2.97%)^b^	25 (31.27 ± 3.03%)^a^	12 (14.59 ± 2.34%)^bc^
D8 blastocysts (% from cleaved)	22 (25.19 ± 3.33%)^b^	31 (37.33 ± 2.16%)^a^	16 (20.25 ± 2.52%)^bc^

*Note*: “*n*” represents the cell number used in this experiment. All experiments were performed in triplicate and the data represent the mean ± SEM. Different superscripts within columns indicate significant differences (*P* < 0.05).

## 4 DISCUSSION

The present study demonstrated that the addition of PCB2 to OMM promoted the development capability of bovine oocytes and improved the quality of embryos. Our results indicated that PCB2 was mainly involved in promoting lipid catabolism via PPARγ/UCP1‐mediated uncoupling, consequently alleviating oocyte oxidative stress and cell apoptosis.

Embryos produced in vitro are more susceptible to ROS attacks than those produced in vivo.^44^, ^45^ Studies have demonstrated that the exogenous addition of antioxidants can effectively improve embryo quality and efficiency of IVP.^46^, ^47^ PCB2, a natural plant extract with powerful antioxidant capacity, showed a much more potent effect on scavenging free radicals and limiting free radical‐induced lipid peroxidation than vitamins C and E.^48^ In the present study, we first found that blastocyst formation and proliferation rates were elevated in bovine oocytes treated with PCB2 (Figure 1A–D, Table 2). Meanwhile, we found that PCB2 significantly increased the ICM/TE rate in blastocysts (Figure 1E,F). Our results showed that PCB2 could alleviate oxidative stress and inhibit oocyte apoptosis during IVM (Figure 2). Since oxidative stress is a disturbed cellular redox state characterized by increased accumulation of ROS.^49^ Accumulating evidence indicates that oxidative stress is one of the main causes of compromised oocyte quality during IVM.^50^, ^51^, ^52^ Our results suggested that PCB2 exerted a beneficial effect via controlling oxidative stress.

Lipid accumulation is a key factor contributing to lipotoxicity‐induced oxidative stress in oocytes.^53^ Thus, we proposed that PCB2 may alleviate oxidative stress by altering the metabolic pattern in oocytes, and our results revealed that PCB2 reduced lipid content in MII oocytes (Figure 3A–E). Our findings were consistent with previous reports that PCB2 treatment could reduce ethanol‐induced lipid accumulation in liver.^54^ Meanwhile, the metabolomic analysis showed that PCB2 mainly affected the uptake of medium‐chain unsaturated fatty acids (C15‐1), and the contents of major fatty acids such as palmitic acid (C16‐0), stearic acid (C18‐0), cis‐9‐octadecenoic acid (C18‐1), and linoleic acid (C18‐2) were decreased in OMM (Table S1). Our results also showed that the expression of genes related to lipolysis and β‐oxidation were increased after PCB2 treatment (Figure 3K). Therefore, we concluded that PCB2 alleviated lipid accumulation primarily by promoting lipid metabolism. Our findings indicated that increased fatty acid metabolism was a protective mechanism for oocytes adaptation to stress environment.

Glucose is a central molecule in many metabolic pathways.^55^ Our results showed that *GLUT1* was markedly reduced (Figure 3K) while the glucose content in OMM was elevated after PCB2 treatment (Table S2), indicating that PCB2 mainly functions in reducing glucose uptake. Similarly, we also found that glucose uptake declined in oocytes after PCB2 treatment (Figure 3F,G). Meanwhile, PCB2 treatment significantly affected the metabolism of Xylitol (Table S2). A previous study showed that xylulose‐5‐P, one of the metabolites of xylitol, could reduce ATP levels by inhibiting glycolysis in the pancreatic β‐cell line.^56^ Moreover, xylitol increased the expression of genes related to fatty acid and uncoupling, suppressing visceral fatty accumulation.^57^ These studies on xylitol further validated our point that the metabolism pattern of oocytes was altered after PCB2 treatment, which exhibited a preference for lipid utilization.

Beyond their role as the powerhouse of the cell, mitochondria are also the central hub for cellular metabolism.^8^ Our results show that PCB2 reduced mitochondrial ultrastructure defects (Figure 4A–F). Interestingly, in the present study, the MMP level and ATP production were decreased in oocytes treated with PCB2 (Figure 4G–I). Notably, mitochondrial thermodynamics also plays a key role in energy balance. Meanwhile, we found that PCB2 could increase mitochondrial temperature (Figure 4J,K). Our previous study suggested that the mitochondrial temperature of oocytes in diabetic mice was reduced, and PCB2 treatment increased the mitochondrial temperature.^16^ SDHB is one of the subunits of mitochondrial respiratory chain complex II.^58^ A previous study showed that succinic acid metabolism could promote cellular thermogenesis.^59^ Similarly, we also found that increased expression of *SDHB* was correlated with elevated mitochondrial temperature (Figure 4L). Furthermore, our results showed that elevated UCP1 expression was accompanied by decreased MMP and ATP contents in PCB2‐treated oocytes (Figure 5A–C), indicating that mitochondrial uncoupling was initiated by PCB2 treatment. Thus, it is not surprising that increased lipolysis was associated with decreased MMP and ATP levels in PCB2‐treated oocytes. Likewise, several studies reported that appropriately increasing mitochondrial uncoupling could effectively alleviate oxidative damage in oocytes.^39^, ^60^ Mitochondrial uncoupling has been suggested as an important mechanism for reducing mitochondrial ROS levels and mitigating cell death.^61^, ^62^ Herein, we also used Mito‐SOX™, a mitochondrial superoxide indicator, to specifically detect the ROS level in mitochondria. Results showed that after PCB2 treatment, the mitochondrial ROS level of oocytes was significantly lower than that of the control group (Figure 5D,E). Subsequently, we further verified whether PCB2 activates UCP1‐mediated mitochondrial uncoupling. Our findings demonstrated that inhibiting UCP1 with GDP resulted in a suppression of mitochondrial uncoupling (Figure 5F–J). Based on the findings of this study, PCB2 promotes lipid catabolism and mitochondrial uncoupling. Consequently, this prompted us to validate the relationship between these two processes. Crucially, our result showed that the reduction in UCP1‐mediated uncoupling corresponded with a decrease in lipid catabolism (Figure 5K,L). To this end, the current results shed new light on the role of PCB2 in reducing oxidative stress and lipid accumulation through UCP1‐mediated mitochondrial uncoupling.

PPARs are ligand‐activated nuclear receptors that regulate glucose and lipid metabolism.^63^ Growing evidence indicates that PPARγ is a potent and protective regulator of lipid deposition.^23^, ^24^, ^25^, ^64^, ^65^ Initially, we observed an increase in PPARγ expression after exposure to PCB2 (Figure 6A,B, S1). Autoduck turned out that PCB2 could bind to the active site of PPARγ, with a binding energy of −8.1 kJ/mol. The result also showed that PCB2 formed hydrogen bonds with PPARγ at GLN454, LYS 458, and TYR477, leading to PPARγ activation (Figure 6C). Our results implied that PCB2 could activate the downstream targets, through the intermediary bridge, PPARγ. Nevertheless, the predictions from molecular docking studies necessitated further validation. Subsequently, we investigated whether inhibiting PPARγ could reverse the protective effects of PCB2. Using the PPARγ antagonist (GW9662), we noted that GW9662 successfully inhibited the lipid catabolism and mitochondrial uncoupling state induced by PCB2 (Figure 6D–I). These findings strongly suggested that lipid catabolism was promoted in a PPARγ‐dependent manner. Furthermore, PPARγ inhibition abolished the beneficial effect of PCB2 on oocyte development (Figure 7A, Table 3). Additionally, the inhibition of PPARγ hindered oxidative stress relief (Figure 7B–E), further reinforcing that PPARγ was the interaction target of PCB2.

In summary, our study revealed that PCB2 improved oocyte developmental competence via PPARγ/UCP1 mediated mitochondrial uncoupling lipolysis and mitigated oxidative stress. These findings will provide novel insights into deciphering the mechanism underlying the regulatory role of cellular metabolism in oocyte development and will be beneficial for the overall improvement of oocyte quality.

Conclusively, explanation of molecular, transcriptomic and metabolomic background determining anti‐apoptotic, anti‐oxidative, catabolic, and lipolytic pathways induced by PCB2 under IVM conditions might give rise to attainment of high parameters of meiotic and developmental competence by bovine and other mammalian IVM‐derived oocytes. The above‐mentioned induction of PCB2‐evoked and PPARγ/UCP1‐dependent intra‐oocyte pathways might promote the propagation and multiplication of excellent‐quality nuclear recipient ova for the purpose of cloning by somatic cell nuclear transfer.^66^, ^67^, ^68^, ^69^ These PCB2‐prompted intracellular networks might also lead to the successful generation of metaphase II‐stage female gametes exhibiting high quality attributes for the purposes of ex vivo fertilization either by non‐assisted monospermic penetration of ova or by microsurgically assisted injection of single spermatozoa into host ooplasm.^70^, ^71^, ^72^, ^73^, ^74^, ^75^

## AUTHOR CONTRIBUTIONS

Jun Li and Xiangwei Fu initiated, organized, and designed the study. Yuwen Luo designed and performed major experiments and analysed data. Lv Zheng, Yizaitiguli Reyimjan, Yan Ma, Shuaixiang Huang, Hongyu Liu, Guizhen Zhou, Jiachen Bai, Yixiao Zhu, Yidan Sun, and Xinhua Zou contributed to the samples collection, performed partial experiment, and analysed data. Yuwen Luo, Jun Li, and Xiangwei Fu wrote the manuscript and Yunpeng Hou revised the manuscript. All authors commented on the manuscript.

## FUNDING INFORMATION

The work was supported by National Key Research and Development Program of China (2021YFD1200402), Key Technologies for Cryopreservation of Gametes and Embryos in Cattle and Sheep (2022BC003), Training and Guiding Outstanding Young and Middle‐aged Talents (SKLSGIHP2021A01), Central Guidance on Local Science and Technology Development Fund (nano‐particles & 226Z7713G), Specific Project of Hebei Province for Outstanding Talents in Clinical Medicine, and Natural Science Foundation of Hebei province (H2023206029).

## CONFLICT OF INTEREST STATEMENT

The authors declare that they have no competing interests.

## Supporting information


**DATA S1:** Supporting Information.

## Data Availability

The data that support the findings of this study are available from the corresponding author upon reasonable request.
