# Supplementary material for: Procyanidin B2 improves developmental capacity of bovine oocytes via promoting PPARγ/UCP1‐mediated uncoupling lipid catabolism during in vitro maturation
Source: Cell Prolif. 2024 Jun 12;57(11):e13687. doi: 10.1111/cpr.13687 (PMC11533046; doi:10.1111/cpr.13687)
Supplement: Supplementary file 1 — DATA S1: Supporting Information. [file CPR-57-e13687-s001.docx]

**Supplementary**


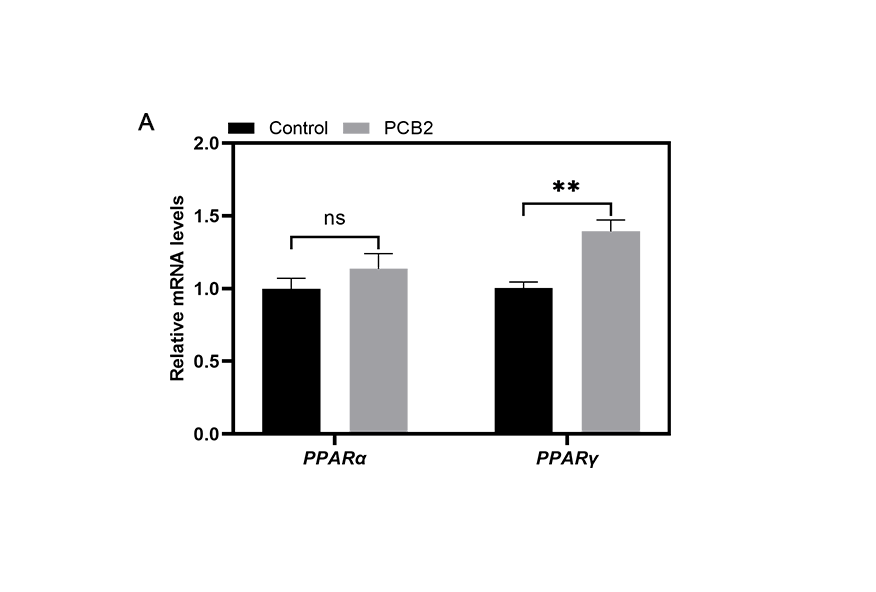


**Figure S1 Statistical analysis of relative expression levels of PPARα and PPARγ in MII oocyte**

(A) Statistical analysis of relative expression levels of *PPARα*, and *PPARγ* in MII bovine oocytes. All experiments were performed in triplicate and the data were represented as mean ± SEM. ns = non significance, ^*^, *P* < 0.05, ^**^, *P* < 0.01, ^***^, *P* < 0.001.

**Table S1 Lipid components detected in OMM and the related metabolic pathways**

| Class | Index | Metabolite | Content (mg/mL) | | Fold change | VIP | Pathway name |
| --- | --- | --- | --- | --- | --- | --- | --- |
|  |  |  | Control (mean ± SD) | PCB2 (mean ± SD) |  |  |  |
| Saturated fatty acid | C9-0 | Nonanoic acid | 0.045 ± 0.066 | 0.037 ± 0.058 | 0.750 | 0.085 | Fatty acid metabolism |
|  | C10-0 | Decanoic acid | 0.731 ± 0.190 | 0.816 ± 0.154 | 1.115 | 0.907 | Fatty acid biosynthesis |
|  | C11-0 | Hendecanoic acid | 0.015 ± 0.025 | 0.040 ± 0.069 | 2.729 | 0.437 | Fatty acid metabolism |
|  | C12-0 | Lauric acid | 0.019 ± 0.033 | 0.027 ± 0.041 | 1.402 | 0.483 | Fatty acid biosynthesis |
|  | C14-0 | Myristic acid | 2.938 ± 0.469 | 3.043 ± 0.414 | 1.036 | 0.581 | Fatty acid biosynthesis |
|  | C15-0 | Pentadecanoic acid | 0.366 ± 0.043 | 0.352 ± 0.048 | 0.962 | 0.335 | Fatty acid metabolism |
|  | C16-0 | Palmitic acid | 271.740 ± 37.413 | 270.700 ± 34.744 | 0.996 | 0.162 | Fatty acid biosynthesis, Fatty acid elongation, Fatty acid degradation, Biosynthesis of unsaturated fatty acids, Fatty acid metabolism |
|  | C17-0 | Heptadecanoic acid | 0.940 ± 0.117 | 0.892 ± 0.0927 | 0.949 | 0.375 | Fatty acid metabolism |
|  | C18-0 | Stearic acid | 181.740 ± 22.360 | 177.830 ± 21.207 | 0.978 | 0.059 | Fatty acid biosynthesis the biosynthesis of unsaturated fatty acids |
|  | C19-0 | Nonadecylic acid | 0.107 ± 0.012 | 0.100 ± 0.011 | 0.935 | 0.268 | Fatty acid metabolism |
|  | C20-0 | Arachidic acid | 1.501 ± 0.147 | 1.390 ± 0.171 | 0.926 | 0.769 | Biosynthesis of unsaturated fatty acids |
|  | C22-0 | Behenic acid | 0.458 ± 0.012 | 0.449 ± 0.0272 | 0.981 | 1.075 | Biosynthesis of unsaturated fatty acids |
| Monouns-aturated fatty acid | **C15-1** | **cis-10-pentadece-noic acid** | **0.382 ± 0.156** | **0.227 ± 0.0597** | **0.593** | **1.823** | **Biosynthesis of unsaturated fatty acids** |
|  | C16-1 | cis-9-palmitoleic acid | 1.849 ± 0.060 | 1.731 ± 0.228 | 0.936 | 0.858 | Fatty acid biosynthesis |
|  | C18-1n9c | cis-9-octadecenoic acid | 7.378 ± 0.442 | 7.035 ± 0.455 | 0.954 | 1.191 | Fatty acid biosynthesis the biosynthesis of unsaturated fatty acids |
|  | C18-1n9t | trans-9-octadecenoic acid | 4.495 ± 0.058 | 4.450 ± 0.148 | 0.990 | 1.371 | Biosynthesis of unsaturated fatty acids |
|  | C19-1(cis-10) | cis-10-carboenoic acid | 4.608 ± 0.295 | 4.31 ± 0.160 | 0.936 | 1.064 | Biosynthesis of unsaturated fatty acids |
|  | C20-1(cis-11) | cis-11-eicosenoic acid | 2.047 ± 0.116 | 1.888 ± 0.206 | 0.923 | 1.542 | Biosynthesis of unsaturated fatty acids |
| Polyunsat-urated fatty acids | C18-2n6t | Linolelaidic acid | 5.765 ± 0.209 | 5.372 ± 0.573 | 0.923 | 1.488 | Biosynthesis of unsaturated fatty acids |
|  | C18-2n6c | Linoleic acid | 1.222 ± 0.051 | 1.128 ± 0.021 | 0.932 | 2.018 | Linoleic acid metabolism, Biosynthesis of unsaturated fatty acids |
|  | C18-3n3 | α-linolenic acid | 1.358 ± 0.049 | 1.360 ± 0.032 | 1.002 | 0.145 | Alpha linolenic acid metabolism, Biosynthesis of unsaturated fatty acids |
|  | C20-3n6 | cis-8,11,14-eicos-atrienoic acid | 4.538 ± 0.040 | 4.521 ± 0.045 | 0.996 | 1.066 | Alpha linolenic acid and linoleic acid metabolism |

The data represent the mean ± SD. Cells highlighted in bold exhibited a significant decrease.

**Table S2 Carbohydrate components detected in OMM and the related metabolic pathways**

| Class | Metabolite | Content (μg/mL) | | Fold change | VIP | Pathway name |
| --- | --- | --- | --- | --- | --- | --- |
|  |  | Control (mean ± SD) | PCB2 (mean ± SD) |  |  |  |
| Disaccharide | Sucrose | 3.6815 ± 0.516 | 3.384 ± 0.069 | 0.919 | 0.873 | Galactose metabolism, Starch and sucrose metabolism |
|  | Trehalose | 0.40015 ± 0.042 | 0.355 ± 0.006 | 0.887 | 1.486 | Starch and sucrose metabolism |
|  | Lactose | 3.8434 ± 0.828 | 3.734 ± 0.338 | 0.971 | 0.133 | Galactose metabolism |
| Monosaccharide | Glucose | 15.047 ± 5.388 | 22.009 ± 14.735 | 1.463 | 0.602 | Glycolysis / Gluconeogenesis, Pentose phosphate pathway, Galactose metabolism, Starch and sucrose metabolism, Amino sugar and nucleotide sugar metabolism |
|  | D-Sorbitol | 2.0561 ± 0.103 | 1.963 ± 0.022 | 0.984 | 1.326 | Fructose and mannose metabolism, Fructose and mannose metabolism |
|  | **Xylitol** | **3.0226 ± 1.076** | **2.270 ± 0.708** | **0.751** | **1.008** | **Pentose and glucuronate interconversions** |
|  | Inositol | 11.413 ± 2.218 | 11.998 ± 1.283 | 1.051 | 0.494 | Galactose metabolism, Inositol phosphate metabolism |
|  | Fructose | 11.433 ± 1.348 | 10.704 ± 1.237 | 0.936 | 0.727 | Fructose and mannose metabolism, Galactose metabolism, Starch and sucrose metabolism |
|  | L-Rhamnose | 2.4204 ± 0.094 | 2.501 ± 0.216 | 1.033 | 0.616 | Fructose and mannose metabolism |
|  | D-Arabinose | 1.7706 ± 0.015 | 1.742 ± 0.014 | 0.955 | 1.651 | Pentose and glucuronate interconversions, Amino sugar, and nucleotide sugar metabolism |

The data represent the mean ± SD. Cells highlighted in bold exhibited a significant decrease.
